# A critical phenomenological investigation in the use of touch as “know how” in practical physiotherapy in primary care with children and adults

**DOI:** 10.3389/fresc.2022.1008969

**Published:** 2022-12-05

**Authors:** Wenche Schrøder Bjorbækmo, Anne Marit Mengshoel

**Affiliations:** ^1^ Department of Rehabilitation Science and Health Technology, Oslo Metropolitan University, Oslo, Norway; ^2^ Department for Interdisciplinary Health Sciences, University of Oslo, Oslo, Norway

**Keywords:** touch, physiotherapy, know-how, phenomenology, children and adults

## Abstract

In this article, we examine the interactions between physiotherapists and patients in actual situations, focusing on how touch is expressed, what it may mean and how physiotherapists know (or do not know) when and how to touch. The empirical material is obtained from two Norwegian research projects. In both of them, the first author observed physiotherapeutic practice and conducted interviews with patients (children and adults) and physiotherapists. A phenomenological research approach was applied, and analysis of the empirical data was guided by the concept of *bridling,* implying adopting a questioning attitude and being open to that which presents itself and exploring its possibilities*.* Three processed excerpts from the empirical data are presented to illustrate how, in different ways, physiotherapists' expert knowledge about how to relate to and interact with individual patients is put into play and expressed in real physiotherapy encounters. Each excerpt is presented individually, followed by analysis. Our findings reveal aspects of the epistemology of physiotherapeutic practice to be intercorporal and illuminated by the concept and phenomenon of *letting the other be*.

## Introduction and background

Touch has played a central role in the history of physiotherapy, whether as part of the physiotherapy examination (palpation) or in specific therapies, such as massage and guided movements (1). In this article, we examine the interactions between physiotherapists and patients in actual practice situations. Our primary focus is on the expression, meaning and significance of physiotherapists' use of touch, understood as embodied knowledge. We explore how touch is expressed and displayed in the physiotherapist's “somatic” or bodily style, behaviour and preferences in practical encounters. In short, we examine some aspects of the epistemology of physiotherapeutic practice, based on an understanding of “practical” behaviour as not being “atheoretical” in the sense of lack of seeing – implying that for action to not be blind, theoretical cognition must be applied (2, p. 69). Throughout this article, we rely on a definition of touch as something about “feeling within, between and across bodies”. This conveys the ambiguity and complexity of touching, which is at the same time physical, affective, literal and metaphorical (3, p. 171).

The phenomenon of touch, while highly present in our lives, is at the same time difficult to capture, given its breadth, depth and complexity. One aspect of touch is that it is absolutely crucial for young animals, as well as human infants. Another is the delicate balance between instances when touch is appropriate and situations when it feels awkward or inappropriate (4). As the American anthropologist Ashley Montague once observed, “We need to understand that we have for too long neglected and overlooked the importance of tactile communication, not only in the development of the infant and child, but also in the development of the adult” (quoted in 4, p. 47).

When touch is viewed as a central element in physiotherapy practice, it becomes necessary to direct attention to the knowledge base and epistemology of physiotherapy practice. Noting that this sphere has received only limited exploration, Edwards and Richardson (5, p. 185) argue that such an epistemology would help legitimise the manifold sources of knowledge underpinning the intersubjectivity of interpersonal interventions in physiotherapeutic practice. Others, too, have argued that practical physiotherapy knowledge (also referred to as “practice epistemologies”) has not been extensively investigated, with the result that tacit assumptions about what counts as physiotherapy knowledge have come to form the basis for many approaches in physiotherapy practice (6, p. 420).

Using the analogy of “bricolage” (the practice of deploying multiple tools, elements and strategies to realize a project), Shaw and Deforge (6) present physiotherapeutic epistemology as an assemblage of multiple types of knowledge. They emphasize the tentative, contextual and dynamic nature of physiotherapy practice and knowledge. Knowledge from a variety of partial perspectives characterizes “physiotherapists as bricoleurs”: practitioners who emphasize the tentative nature of their knowledge and recognize the roles of history, society, and power in creating and changing what they know. Shaw and Deforge advocate drawing on bodies of knowledge that are undervalued and marginalized, and encourage physiotherapists to explore new and varied ways to approach physiotherapy practice. On this basis, they argue, the profession can progress to a more holistic understanding of how physiotherapy may contribute to people's health and well-being (6, p. 427).

We see our research into the nature of embodied, practical physiotherapy knowledge and its possible forms of expression in real-life situations as a contribution to this larger project.

Given that our specific focus is on the phenomenon of touch, what does the existing literature have to tell us about this phenomenon, its nature and uses? In 2002, Roger et al. (7) found that, despite touch being a basic element in the practice of physiotherapy, little research had been done regarding its forms and purposes. For their research, they videotaped 15 physiotherapists treating 2–3 patients each before reviewing the videos of themselves in action and describing the types of touch and the intent behind their use of touch. Their findings shed light on the most common types of touch used in physiotherapy. The participating physiotherapists were found to use 33 different combinations of touch – that is, a single touch was used for more than one purpose. They concluded that physiotherapists clearly performed in a “high-touch” arena (7).

More recently, physiotherapy researchers have begun to pay more attention to the phenomenon of touch and have done so in various ways. In their research, Hiller, Delany and Guillemin (8) showed how touch was used to demonstrate care, empathy, support and reassurance, as well as for communicative purposes. However, the participating physiotherapists rarely described touch as an explicit form of communication. In contrast, the participating patients expressed how their physiotherapists' touch represented care and built their confidence in the physiotherapists.

In a previous exploration of how touch is used and expressed in physiotherapeutic practice, Bjorbækmo and Mengshoel (9) found that it resembled an embodied intercorporal dialogue between the patient and the physiotherapist, similar to the way two bodies relate to one other in dance (9).

On the basis of their research, Geri et al. (10) argued for a change in perspective within manual therapy regarding the use of hands-on techniques, and recommended further research on the multifaceted mechanisms of these practical techniques (10).

Kelly et al. (11) used a meta-ethnography approach to synthesise a coherent conceptualisation of touch across health disciplines in order to inform and support interdisciplinary praxis of touch in healthcare. Their search of 8 databases identified 41 studies involving 7 professions; significantly, of this total only 5 studies were from the field of physiotherapy. Their findings revealed that while different health professions expressed care through touch in different ways, all professionals expected themselves, rather than patients, to be the ones to initiate touch (11).

In their study based on enactive theory, Sørvoll et al. (12) explored touch in paediatric physiotherapy. They found that touch, understood as comprising both physical and social elements, blends with paediatric physiotherapy through co-regulative interaction processes. The authors highlighted how the many modalities of touch were significant in all clinical encounters, whether those involving infants, children, adolescents or adults (12).

Other researchers have investigated the knowledge base of physiotherapy. Supported by phenomenological philosophy and enactive theory, Halak and Kriz (13) argue that physiotherapy is about physiotherapists' empathy with patients' bodily intentionality and that this involves reciprocal coordination and open-ended bodily dialogue, similar to that found in the context of dance. They suggest that along with using language to explain and apply theoretical knowledge of various kinds, physiotherapists should build on the reality of their own embodiedness and their previously acquired practical and bodily knowledge (13).

The focus of our research is how touch is expressed in interactions between physiotherapists and patients. How do physiotherapists know (or not know) when and how to touch? What means are associated with, or seem to result from, different forms of touch?.

In the next section, we set out our theoretical framework and describe the methodological approach adopted in the two projects from which are the source of our empirical material. Thereafter three examples from the empirical material are presented individually, each followed by analysis. In the final section, we discuss the significance of our findings and provide suggestions for further research.

## Theoretical perspective

We start by explaining phenomenological perspectives on the body, tactility and touch. Then we give a brief account of tactile therapies, including physiotherapy, before elaborating on different concepts and understandings of embodied knowledge.

### Phenomenological perspectives on body, tactility and touch

Informed and inspired by Maxine Sheets-Johnson (14), we regard all humans – whether children or adults, patients or therapists – as tactile-kinaesthetic, affective bodies whose social sense-making is foundationally intercorporally anchored. As bodies, we are attuned to our own felt dynamics and literally and metaphorically feel our way in a shared interworld. As living, animated bodies, we are primed by our bodily surfaces and organs for tactile experience (14). Tactile experience is achieved through the interactions of all senses, opening a world to us. All experiences and perceptions thus involve syntheses of multiple sensations, with many forms of possible interplay among them. This raises the possibility of seeing sounds and hearing colours (15) or seeing with the hands and touching with the eyes (3).

The physiology of touch embraces the tactility of the skin; the flesh, with its deeper, more muscular feelings of movement; and the body as a somatic set of sensations (3, p. 79). The manifold meanings of touch reveal themselves the more deeply a researcher delves into the physiology, psychology and fleshy philosophy of the body. From a physiological perspective, touch can be described as a modality that results from the combined information of innumerable receptors and nerve endings concerned with pressure, temperature, pain and movement.

However, touch is also a way of communicating empathy, love, desire, punishment or disgust; it has the capacity to bring objects and people into proximity in various ways (3, p. 1). Touch is also the first sense to develop in the human embryo. Yet despite being crucial to our embodied existence, touch remains an under-examined component of everyday experience, scarcely discussed and largely neglected and forgotten (3, p. 2–5).

### Tactile therapy

Tactile therapies of various forms have long stressed the ability of touch to heal and cure. While this evokes mystical associations, Classen (16) argues that there are in fact two distinct streams in the history of therapeutic touch: first, the supernatural stream, which she refers to as the “royal touch”, and second, the stream focussing on the natural healing powers of touch, as in physiotherapy. In non-Western societies, such distinctions between “supernatural” and “natural” seemed to have been less pronounced, particularly in the pre-modern period (16, p. 348).

In physiotherapy, there has been a strong tendency to resort to the body-as-machine metaphor. Where an individual's physical body is essentially understood as a (functioning or non-functioning) machine and touch is limited to measuring, diagnosing and fixing what is not working (1). In the case of massage, the objective is understood to be to fix circulation issues and/or ease tension in the “body-machine”. This understanding of the body tend to obscure, forget and not recognising the healing and curative properties of touch (16, p. 348). However, therapists have long realised that interacting with their patients' bodies demands much more than simply viewing the body as a machine.

We understand professional practice in medicine, in the health sciences and specifically in physiotherapy as essentially interpersonal and constituted *in between* the patient and the therapist in the present moment (17). The outcome of a therapeutic encounter is never predictable, no matter how much care is invested in planning examinations and treatments. While therapy may have one or more goals, the path to achieve the desired outcome can never be precisely determined. There is always an element of openness, in terms of both outcome and chosen path. Therapists must, to a certain degree, venture into the unknown. They must prepare themselves for what has not yet occurred; they must endure and live with uncertainty and “not knowing” (18).

### “Know-how” vs. “know-that”

The British philosopher Gilbert Ryle (19) has explored the tendency to perceive intelligence as tied to the exercise of specific internal acts (acts of thinking). He argues that practical activities are only described as intelligent or “clever” when they are accompanied by some internal acts of considering propositions (particularly “regulative” propositions). Doing things is thus never in itself perceived as an exercise of intelligence; at best, “doing” is a process introduced and somehow steered by some ulterior act of theorising. Theorising is thereby not regarded as a form of doing, setting up a contradiction between “internal doing” and “external doing”.

In fact, Ryle (19) here addresses the well-known gap between theory and practice. To act and to perform something requires a certain style, method or modus operandi. Doing something (whether internally or externally) intelligently does not involve doing two things – one in the doer's head and the other in the actual world: it entails doing one thing in a certain manner. It is about embodied, embedded and enacted know-how; to this extent, it is akin to dancing (19).

In general, philosophers have done insufficient justice to the distinction (quite familiar to most of us) between knowing that something is the case and knowing how to do things. In their theories of knowledge, they have either concentrated on the discovery of truths/facts or ignored the discovery of ways and methods of doing things. In a departure from this, Ryle (19) argues that knowledge about “how” cannot be defined in terms of knowledge that something “is”. Furthermore, knowledge-how is a concept that is logically prior to the concept of knowledge-that. In other words, it requires intelligence, not only to discover truths but also to apply them.

For Ryle, an important point is that knowing how to apply truths cannot be reduced to knowing that something “is like it is” (knowledge of facts). With reference to Ryle, Brandt (20) argues that knowledge-how is not merely a capacity to get things right; it is a multi-track ability, a capacity to get a variety of things right. Unlike knowing how to do a single type of thing, knowledge-how is exercised in relation to different acts. For example, knowledge of how to manage a company involves an infinite variety of acts, not just a single type of act (20).

The complexities and subtleties of human knowledge demand what De Jaegher (21, p. 853) calls “high-level practical connecting know-how”. For her, this kind of knowing needs to be viewed with fresh eyes, with attention paid to how to account for it. Gaining a better understanding of human knowing has important implications for how we treat one another. In the drive to understand higher intelligence, this kind of sophisticated knowing has for long remained out of cognitive science's purview, suggesting that a wealth of human knowing has been overlooked. Such knowledge lies just beneath our noses: for instance, “knowing what is going on with someone from seeing the way they lift their gaze” or “how to make ideas felt in poetry” (21, p. 848). Such sophisticated knowing is always characterised by uncertainty, inconsistency, ambiguity and contradiction; it is how we routinely deal with ourselves, one another and the world around us.

De Jaegher (21) argues that the enactive theory of intersubjectivity as participatory sense-making, with its dialectical approach to how people co-make sense in moving, breathing and living together, goes some way towards explaining human knowing. At the same time she senses that something is missing – a certain depth in the area of epistemology that she calls “letting be”, described as a deep form of engaging, of relating between parties interested in knowing each other (21, p. 849).

## Method

In both the research projects that yielded the data analysed and discussed in this article, a phenomenological theoretical approach was used to explore the notion of touch and embodied, embedded practical knowledge. For Merleau-Ponty (15, p. viii), “phenomenological insight is only accessible through a phenomenological method”. Such a phenomenological method aims to break through and gain access to pre-reflective experiences as they occur in taken-for-granted situations and activities (22, p. 215).

In one of the above-mentioned research projects, data was generated from interviews with 23 children (aged 4-12), diagnosed as having either serious congenital heart disorders (indicating that they had undergone a surgical procedure involving multiple and complex corrections during their first year of life) or a motor function disability. Additional data was derived from observations of seven of the children at one of their weekly physiotherapy sessions.

In the other research project, the material was generated from 16 close observations and interviews with 9 physiotherapists and 9 adult persons suffering from long-lasting neck pain (defined as lasting for more than three months).

For van Manen (22, p. 318, 23, p. 69), close observation involves an “attitude of assuming a relation that is as close as possible while retaining a hermeneutic alertness to the situations that allows us to constantly step back and reflect on the meaning of those situations”. Applied to the two research projects cited here, close observation enabled the researcher to relate to physiotherapists and patients by being with them, patiently waiting, not attempting to participate in what they were occupied with, but also willingly accepting their invitations to listen and talk with them.

The role of the qualitative researcher as co-creator of the generated data is now well established (24). In the context of this research, we understand co-creation in a research encounter as being about each person (patient, physiotherapist and researcher) touching and impacting the other, thereby shaping how the research unfolds and the characteristics of the generated data (25, 26).

All interviews were audio recorded and transcribed by the first author, who also conducted the observations, writing field notes immediately after each one. Prior to the observations of physiotherapy encounters with *child participants*, the first author conducted interviews with the children and their parents in their own home settings. This gave children and their parents the opportunity to get to know the researcher and decide whether they would allow her to do an observation or not. For the study that included *adult participants*, all observations were made prior to the interviews with both patient and physiotherapist. This choice was made so as to allow the researcher to raise questions during interviews regarding aspects of what she had observed. Interviews took place in a quiet room at the physiotherapy clinic shortly after the observations.

For both studies, the primary approach (in respect of planning, conducting interviews and observations, analysing and presenting the findings) was a phenomenological one centred on asking questions (23, 27). Dahlberg and Dahlberg (27, p. 891) refer to Merleau-Ponty's understanding of the question as a “way of knowing and asking at the same time”. This phenomenological attitude is about questioning that which we already know something about. It is about looking again, observing with fresh eyes what we take for granted. Since we generally live in the world we are studying, we need to question it to see it anew (27, p. 892).

Dahlberg and Ekebergh (27, 28, 29) have proposed the concept of *bridling* as an approach to examining and questioning empirical researchers' meaningful relationship with the world they inhabit and study. The practice of bridling involves adopting a questioning attitude, being open to that which presents itself and exploring its possibilities. It entails being attentive to what we hear, see and understand without immediately being certain of the meaning of that which we have heard, seen and understood. It is not about understanding too quickly but rather questioning that which we have understood. Such an openness can be compared to a form of improvisation in which the researcher cannot be certain of what will turn up or show itself but has to be attentive and ready for it (27, p. 894).

Bridling involves the shift from a natural attitude (the taken-for-granted) to a phenomenological attitude of openness and questioning (29). Throughout our research, we sought to make bridling central to our efforts. We strove to maintain a questioning attitude by dwelling on what was said in interviews or enacted during observations. We tried to avoid jumping to conclusions. In our analyses of the data from the two studies, we aimed to stay open to what we might see and hear, dwelling with physiotherapy as an intersubjective, intercorporal, embodied, embedded and enacted professional practice.

Following Dahlberg and Dahlberg's (28) notion of the third way of conducting an analysis, our analysis consists of two parts.

The first part (or stage) deals primarily with the presented empirical material. While recognizing that we can never be atheoretical, we here seek to focus on, and dwell with, what we have heard and seen. We ask what the participants are doing when they do what they do and what they are saying when they say what they say.

The second part entails applying the lens of theory in a bid to illuminate further aspects of the examined phenomenon: aspects that remain partially obscured or difficult to perceive. The theory in question is one that is sensitive to the phenomenon and compatible with our commitment to bridling, to staying open to the meanings of the phenomenon of touch and know-how in the practice of physiotherapy (28).

By applying this two-step approach to analysis, we hope to have coaxed out some neglected or overlooked aspects of the phenomenon of touch in the lifeworld of physiotherapists and their patients.

During the process of analysis, we collaboratively reflected on and discussed specific extracts from the material particularly relevant to our focus on the phenomenon of touch. In addition, the first author wrote (and rewrote) the examples based on field notes and transcriptions from the interviews, always seeking to stay as close as possible to the experiences that unfolded during observations and interviews. Following several rewritings and discussions of the examples, we eventually decided which examples best illustrated touch in physiotherapy practice as a taken-for-granted and complex phenomenon. By including examples from physiotherapy practice with both children and with adult patients, we have sought to bring out similarities and differences between physiotherapy practice in respect of these two categories.

Both research projects were undertaken according to the Helsinki Declaration and approved by the Regional Committee for Medical and Health Research Ethics in Norway (Study 1, ID 2011/48 and study 2, ID 2012/174). Information on the project and the consent form were available in Norwegian. All patients (children and adults) were provided with written informed consent forms. Since the children were all below the age of 16, consent was given by their parents. Children who were able to write their own names also provided written assent.

## Findings

The three examples presented below have been selected because in different ways, they show how physiotherapists' expert knowledge of touch and how to relate to and interact with individual patients is put into play and expressed in real physiotherapy encounters. Example 1 comes from the first of the two studies, in which children were the patient-participants. Examples 2 and 3 are taken from the second study, with its adult participants; the same physiotherapist features in both examples. These examples have been chosen to illustrate the similarities and possible differences between physiotherapy for children and for adults, as well as to illustrate how a physiotherapist applies different approaches to different patients, despite their having similar diagnoses.

### Example 1

Peter, aged 10, has a diagnosis of severe congenital heart disease. He arrives at the physiotherapy clinic together with an assistant who has driven him here from school. The assistant must wait for him in the reception area. Peter enters the physiotherapy gym with Jenny, his physiotherapist.

*Peter walks straight to the trampoline and starts jumping. Jenny, the physiotherapist, follows him. While standing next to the trampoline, she tells him about a girl who earlier this day had managed to get across the floor of the gym by walking on a big bolster. Peter goes to the treadmill and starts walking. Jenny follows him and regulates the resistance on the treadmill. He also regulates the resistance. He alternates between walking and running. The running stages are shorter than the walking stages. He finishes on the treadmill. Taking a short break, he stands close to Jenny, who strokes his back and says (addressing me, the researcher), “Sometimes he gets a massage, gets some cuddles.” Peter slaps his hand on his forehead, rolls his eyes and bows his head, looking down at the floor. Nothing more is said about massage or cuddling. Peter does several activities, and after a while, he and Jenny agree that he should try the bolster (approximately 80 cm in diameter). On all fours, he climbs on top of the bolster; he then gets to his feet but soon after, gets down on his hands and knees again. On top of the bolster, he moves his hands and legs and moves forward, while Jenny walks beside him. They continue crossing the floor in their own ways, calmly talking together*.

*During his interview the day before the observation, Peter had said, “Sometimes if I go to the gym in school, I get a massage by the physiotherapist…. It*”*s really nice….”*

At the start of the session, the physiotherapist seems sensitive to Peter and his reactions. She waits and does not push him to try to walk on the bolster. She provides him with time, space and possibilities for action when she follows his choice of which activity to start with. During the break when she strokes and pats him on the back, this seems to be something that he accepts, but when she uses the word “cuddles” in the context of a massage, a change occurs. Still standing close to the therapist and almost leaning against her, Peter uses gestures to indicate that he finds what she has just said embarrassing. During his earlier interview, he had mentioned how he sometimes got a massage and experienced it as “nice”.

Peter's reaction carries a clear message, to which Jenny responds by tacitly accepting his expressed embarrassment and letting things be. She continues the session, which ends up with Peter carefully crossing the floor on top of the bolster, with the physiotherapist walking beside him.

What seems at stake here is Peter's understanding of the terms “massage” and “cuddles” and the possible relation between them. He may associate massage with something other than the cosiness of “cuddles”, even though he finds it “nice”.

The word “massage”,^1^ derived from Greek, means kneading or pressing. It is associated with therapeutic techniques of touching intended for healing and curing, with the promotion not of pleasure or enjoyment but rather of an effective improvement in the person being massaged (30). It may be such an understanding that Peter perceives as the acceptable one; as he understands it, comfort, cosiness and enjoyment are not what massage is all about. It could also be that he perceives Jenny's comment as positioning him as a small child who needs a little cuddle. Or it might be a combination of these understandings that Peter opposes with his gestures. He does not want to be placed in the position of a small child – or as someone who does not understand what massage is about.

This places the therapeutic function of massage somewhere between effect and pleasure and maybe even at the intersection of pain and pleasure. Another point is that while adult patients also receive massages, it would be very unlikely for the therapist to use the word “cuddles” with them. “Cuddle” and “massage” are clearly words that carry certain meanings. Touch in the form of massage is understood in certain ways, which vary according to a number of factors, including culture, the specific social setting and the ages of those involved.

Another point of interest in this example is the physiotherapist's response to Peter's expressed embarrassment. Rather than trying to cover up, joke about it or explain, she simply lets the situation rest. The interrelation between them seems to make her understand, accept and respect his response to her utterance. The embodied, participatory sense-making between them suggests that Jenny senses and uses what De Jaegher calls “letting be; letting the other be” – high-level practical know-how (21). “Letting it be” is not about the absence of care or concern; rather, it is a sensitive and emphatic way of handling tensions between the knower's being (the physiotherapist) and the being of the known, in this case, Peter and his gestures expressing embarrassment at Jenny's comment.

De Jaegher (21, p. 849) argues that knowing-in-connection is the type of knowledge that can be expressed as let it be; let the other be. She refers to the philosopher Kym Maclaren as the one who introduced the concept of letting be, “letting the other be” (31). Maclaren argues that intersubjectivity is given to us in a corporal manner through others' actions. It means that the other's action, in this case, Peter's gesture, bodily implicates the physiotherapist and situates her in a certain way as a self (31, p. 189). The challenge of “letting the other be” is that it requires mutual letting be (31, p. 197). Others̕ actions never simply “let us be” or leave us free to be who we are because we are always situating and determining one another through our own actions. What Jenny and Peter say and do will constantly involve the positioning of each other. This positioning can turn into a battle between the parties, whose collaboration breaks down, or one or the other can give up and let the other take the lead. Alternatively, it can become an interaction where both parties give and take time and space so that they coexist in ways that allow both to let the other be, without having to surrender and conform to the other's understanding and perception of the situation or initiate a fight to decide which of them has the right perception. Letting the other be makes room for different understandings and perceptions, enabling people to live side by side in an encounter in a way that Merleau-Ponty describes as co-existing-subjectivity (15, 32). However, these attitudes and this knowledge must be constantly negotiated, restored, refined and further developed.

In Example 1, this is what Jenny can be understood as practising: let it be; let Peter be. Since this requires a joint effort, Peter shows that he also lets Jenny be, although she has said something that he perceives as embarrassing and perhaps even hurtful. De Jaegher (21) refers to lovers as those who can most clearly show this ability to let the other be. Physiotherapy may seem to involve letting the other be, whether the patient is a child, a youth or an adult. This requires the physiotherapist to know when and how to touch or not to touch, to talk about touching, and how to get in touch and keep in touch with the patient.

### Example 2

John, an experienced physiotherapist, meets Vivian, his patient, for their third appointment. Vivian is in her sixties and is suffering from chronic neck pain.

*Vivian is lying on her back on the treatment table. John stands at the head of the table, holding her head in his hands. Vivian closes her eyes. John bends his head a bit to the right, gazing out into space. There are sounds of his hands against the treatment table, moving her head from side to side, and of her body moving on and against the treatment table. The movements become faster. John's moving of Vivian's head to one side is followed by a quick return to the centre. After a while, they do the same movements on the other side. John rolls on his feet at a standstill, walking-in-place motion. Vivian's arms and hands slide up and down in harmony with her head-turning movements. She closes her left hand – soon after, she opens it again*.

*I interview Vivian right after the treatment session. Remembering the closing of her left hand, I ask if she would say something out loud if something were uncomfortable or painful during the treatment. “I do not have to. It is not necessary…he understands,” she answers*.

John touches Vivian's head with his hands while gently holding the back of her head and, in a way, embracing it. She lets him touch her head and allows herself to let it rest in his hands. They both cooperate and participate in the head-and-neck movements in what appears to be a cohesive way. What then happens between them when Vivian closes her left hand and soon after opens it again?.

John and Vivian show a focused mutual awareness, where both seem highly present in terms of an embodied intercorporal communication. Haptic touch is obvious and present in this example. As Paterson (3, p. 101) notes, it is through haptic experiences that we feel engaged with the world, and through which the world, its objects and others touch us. It is through our intercorporality, our bodily perception of another body – a perception in the form of a bodily resuming of an intentionality we inhabit over there – that we have some possibility of inhabiting the other's perception of the situation in which we are both involved (31, p. 190). When Vivian closes her left hand, it is an expression of something. We interpret John as perceiving this, since Vivian soon after opens her hand again. The closing of her hand may be understood as a sign that something is painful, uncomfortable or not acceptable to her. The fact that she opens her hand again, and her comment during the interview that she has no need to say anything because John understands how the treatment is for her, illuminates how, through touch and movement, they perceive each other's intentionality. They understand each other at an intercorporal level. The expressed silent-touching motion between them is about this intercorporal communication and sense-making, leading on to the expressed, dance-like collaborative motions.

This is an expression of high-level practical know-how (21) executed by the physiotherapist. Such knowledge relates to “letting the other be”, as expressed through coordinated bodily attunement between interacting body-subjects, directed towards and by each other in a vigilant manner.

#### Example 3

John, the same physiotherapist, and Christina, who is in her thirties and suffers from chronic pain in her neck and head, meet for their fourth appointment.

*Christina lies on her right side on the treatment table. She has her right arm under her head on top of the pillow. Both her knees are bent. Her left forearm rests along John's left forearm, so that her whole arm, from elbow to wrist, is supported by his forearm. With his right hand, John touches Christina's shoulder and moves it back and forth. After a while, the shoulder movements go up and down, then shortly afterwards, there's a switch to a circular motion. John asks Christina to participate in doing the movements. “I”m not relaxing enough today,” she says. “I think you are doing well,” he replies. “Do you?” she responds. “If you are tense, I would recognise it immediately,” he assures her. “Would you?” she asks. “Yes, immediately,” he replies*.

*After this session, during my interview with John, he tells me, “I feel I have to be very careful and particularly sensitive with Christina… there is something….”*

The touching between John and Christina differs from that during John's session with Vivian. The embodied knowledge and bodily interrelations put into play seem to make John maintain more physical space between himself and Christina. He supports their interbodily enactment by using words to explain his intentions and how he understands Christina's participation in what they are doing together*.* When in the interview afterwards, John says that he feels that he has to be sensitive and careful and “there is something”, this felt knowledge seems important for his enactment and for their embodied interrelation during the session.

With reference to Howes (33) and to the phenomenon of skinscapes,^2^ John can be understood as using his “skin knowledge”: tactile knowledge that involves the intelligence of the sentient body. When John assures Christina that he would immediately notice it if she were tense, he asserts his confidence in his ability to pick up on his patient's bodily response, on the expression of what is happening there and then between them.

John makes use of his skin knowledge, a tactile sensitivity that tells him how it is for the other without recourse to words. This skill derives from the phenomenon that the one who touches benefits from it by being touched back. Through embodied sensitivity and awareness, the one who touches receives knowledge that is conveyed through the other's body and bodily responses. This tells the one who touches how the touching between them is perceived by the other (30, p. 365). However, this intercorporality, this shadowing of the other's intentionality, seems less clear and obvious to John as the interbodily resonance he experienced with Vivian in example 2. In contrast to John and Vivian's mutual understanding, John is not fully attuned to Christina's perception of the situation in which they are both involved.

In example 2, Vivian clearly expresses her awareness of the physiotherapist's understanding of how it is for her to be touched and moved during their interaction. However, John seems to struggle with establishing bodily dialogue and harmony with Christina. His use of words seems to express his intent to convince Christina that he understands her. When Christina says that she is not sufficiently relaxed that day, John replies that he thinks that she is doing well and that he would feel it right away if she were tense. Does this indicated that he is positioning himself as the “knower”, while positioning Christina as the one who does not know and understand that well?.

According to Maclaren (31), all actions have multiple intentionalities, which means that we always inevitably assert not only ourselves but also the positioning of the other in relation to ourselves when we act (through words, touch, movement or other types of action). This means that others' actions always situate and determine us, just as we, through our actions, always situate and determine others' positioning in relation to ourselves.

Through the three presented examples and the subsequent analyses, we have examined the interaction between physiotherapist and patient in real situations. Our focus has been on how touch is expressed and displayed in the physiotherapist's somatic or bodily style and behaviour in practical encounters. Our findings have revealed aspects of the epistemology of physiotherapeutic practice to be intercorporal, and to involve the concept and phenomenon of “letting the other be”.

## Comprehensive reflections

In physiotherapy, the use of touch is a broad-based phenomenon, involving haptic touch as cutaneous and always working in conjunction with proprioception, kinaesthesia and the vestibular sense (3, p. 15). Our findings illuminate how the combination of these somatic senses in the practice of physiotherapy opens the possibility for an embodied and embedded intercorporal communication between physiotherapist and patient. This intercorporal experience relates to what Paterson (3, p. 15) calls “felt phenomenology”, where flesh (including skin) is the medium rather than an organ. At the same time, Paterson (referencing Aristotle) argues that “if the sense of touch corresponds to any particular organ it would be the heart” (3, p. 17). One way or another, the sense of touch touches our hearts.

Our findings reveal how the intercorporal experience of a shared situation in the practice of physiotherapy always comes about when physiotherapist and patient (whether child or adult) perceiving both participants as subjects who are simultaneously related to each other. This perception of the other “as communication and solitude, as with me and other to me” (31, p. 189) does not involve an either-or choice; rather, it represents two aspects of one phenomenon. All actions have multiple intentionalities (31), which means that in the practice of physiotherapy, the therapist asserts not only their own self but also the positioning of the other (the patient) in relation to “oneself as therapist”. Hence, any action in the shared context of physiotherapeutic practice needs to be contextualized within the place occupied by patient and physiotherapist in relation to each other during an encounter.

Such mutual positioning takes subtle, discreet forms that can be difficult to discern (31). As Maclaren explains, others' actions act on us, not simply by imposing a meaning on us that we can choose to either resist or accept, but by implicating us in this positioning of ourselves, so that we find ourselves already occupying a certain position in reality and having no choice but to deal with it. In this sense, others' actions never simply “let us be” or leave us free to be who we are (31, p. 196).

While this implies that physiotherapists’ actions always situate and determine their patients (children as well as adults), patients also situate and determine their physiotherapists through their way of acting.

Maclaren describes letting the other be as something that must be learned at a corporal level. To achieve letting the other be is a process propelled by both the particular way that each individual is intertwined with others and the implicit metaphysics that each individual has come to enact in one's bodily taking up of oneself, the others, things and the world (31, p. 188). Through such a process and in their practice, physiotherapists can come to understand and acknowledge that in every patient encounter, they and their patient are intertwined and co-determined with and by each other.

Approaching every physiotherapy encounter from such an understanding opens way for therapists to gain knowledge about themselves as well as the patient in the situation. For this to happen, a process of corporal and intercorporal learning must take place during each encounter. For the physiotherapist, such learning always involves some kind of struggle, some degree of effort to let each particular patient (whether child or adult) be, while at the same time finding one's own “free space” (31, p. 188) in the interaction. As Maclaren puts it, “The happy miracle of being with others is that their modes of intending the world can open us to new meanings” (31, p. 191).

On the basis of our research, we argue that, for physiotherapists, “expert knowledge” involves acknowledging the power, complexity and multiple expressions of touch. Touching, being touched and getting, being and staying in touch are dynamically created, developed and expressed in situations where the goal might be known, but the path to achieve it remains open and uncertain.

We recommend further research aimed at exploring the phenomenon of *letting the other be* as a central element in the practice of physiotherapy, and one necessary for the development of the profession.

## Data Availability

The raw data supporting the conclusions of this article will be made available by the authors, without undue reservation.

## Footnotes

^1^Massage for healing had great importance in ancient times when science and technology were not highly developed. In those times, ancient Indian surgeons and physicians studied the art of massage from the famous books of Charaka, Ashtanga, Hridaya and Sushruta. In the 19^th^ century, the Swedish doctor Per Henrik Ling developed what is now known as Swedish massage. By synthesising his knowledge of gymnastics, physiology and Chinese, Egyptian, Greek and Roman techniques, he developed the Swedish massage system.

^2^Skinscapes or *Ichi una* (the term used by the Cashinahua of Eastern Peru) is the knowledge of the world that one acquires through one’s skin – the feel of the sun, the wind, the rain, the forest, and so on (see 28, p. 27).
